# 0521. A functional citrulline-arginine-no pathway and nos3 complex is essential to maintain microcirculatory function during endotoxemia

**DOI:** 10.1186/2197-425X-2-S1-P30

**Published:** 2014-09-26

**Authors:** KAP Wijnands, DM Meesters, B Vandendriessche, JJ Briedé, BAP Bessems, HMH van Eijk, P Brouckaert, WAP Buurman, A Cauwels, M Poeze

**Affiliations:** Maastricht University Medical Center, Department of Surgery, Maastricht, Netherlands; Ghent University, Inflammation Research Center, VIB, Ghent, Belgium; Maastricht University, Maastricht, Netherlands; Maastricht University, Department of Surgery, Maastricht, Netherlands

## Introduction

Sepsis is associated with high mortality rates as a result of the development of multiple organ failure (MOF). In MOF the competition for the amino acid arginine between the microcirculation and the inflammatory response is important. The endothelial nitric-oxide synthase (eNOS) uses arginine to produce nitric oxide (NO), which results in microcirculatory vasodilatation, whereas arginase and the inflammatory nitric-oxide synthase (iNOS) use arginine in the inflammatory response to produce acute phase proteins and to stimulate the host response. Enhanced arginine consumption combined with decreased arginine *de novo* synthesis from citrulline results in arginine deficiency for eNOS, eNOS uncoupling, and a decreased organ perfusion.

## Objectives

To investigate the role of the NOS enzymes during endotoxemia and the effect of citrulline supplementation on the arginine-NO synthesis and microcirculation.

## Methods

Wildtype (n= 39), deficient iNOS mice, (iNOS^-/-^; n= 39), deficient eNOS mice (eNOS^-/-^; n= 39) and double knock-out (KO) mice (mice deficient for iNOS and eNOS; n=39) received a continuous LPS (200µg total) infusion for 18 hrs alone or combined with citrulline (37.5mg) for the last 6 hrs. SDF-imaging was used to evaluate the microcirculation in jejunal villi and the NO production in jejunal tissue was determined by *in vivo* NO spin trapping and quantified by electron spin-resonance spectrometry. After the microcirculatory measurements or *in vivo* NO spin trapping, mice were sacrificed, blood and tissues were sampled. Amino-acid concentrations in blood and tissue were measured by High Performance Liquid Chromotography.

## Results

LPS infusion significantly decreased plasma arginine availability in all mouse strains. However, in the jejunal tissue of iNOS^-/-^ or eNOS^-/-^ mice this was not accompanied by a decreased intracellular arginine availability. Jejunal NO production was significantly decreased in all mouse strains during endotoxemia, except for eNOS^-/-^ mice. LPS infusion resulted in a significantly decrease in jejunal microcirculation in wildtype and iNOS^-/-^ mice. However, mice lacking a functional eNOS enzyme, as present in eNOS^-/-^ and double KO mice, perfusion in jejunal tissue did not decrease during LPS infusion. Also the beneficial effects of citrulline supplementation during LPS infusion on the microcirculation were not present in eNOS^-/-^ or double KO mice, although citrulline significantly enhanced the plasma arginine availability in all mouse strains. In addition, tissue arginine availability did not increase in citrulline supplemented NOS deficient mice.

## Conclusions

Citrulline improves the arginine *de novo* synthesis during sepsis, however to enhance the microcirculation during sepsis, citrulline requires a functional eNOS enzyme. Therefore, future research needs to focus both on improvement of the arginine *de novo* synthesis and maintenance of a functional eNOS enzyme during sepsis.

